# The 5:2 diet induces male-specific weight loss and sex-independent enhancement of lipid storage in mice via activation of GHSR

**DOI:** 10.3389/fnut.2026.1735869

**Published:** 2026-05-14

**Authors:** Amanda K. E. Hornsby, Oscar D. T. Powell, Irina A. Guschina, Taylor J. Williams, Katie D. Lines, Luke D. Roberts, Jeffrey S. Davies, Timothy Wells

**Affiliations:** 1 School of Biosciences, Cardiff University, Cardiff, United Kingdom; 2 Molecular Neurobiology, Institute of Life Sciences, School of Medicine, Swansea University, Swansea, United Kingdom

**Keywords:** 5:2 diet, ghrelin, GHSR, growth, intermittent fasting, lipid storage, metabolism, sexually dimorphic responses

## Abstract

**Introduction:**

Despite its popularity and reported benefits in humans, preclinical characterization of the broader physiological impact of the 5:2 diet and potential mechanisms of action are lacking.

**Methods:**

To address this deficit, male and female wild-type (WT) mice and mice lacking the receptor for ghrelin (GHSR) were subjected to the 5:2 diet for 3 or 6 weeks with growth and metabolic outcomes assessed.

**Results:**

The 5:2 diet reduced weight gain in adolescent male mice by 31% and induced minor weight loss in adult males without affecting body weight in females. Tibial growth rate in adolescent male and female mice was elevated by 9–13%, with marrow adiposity doubled in adolescent males and halved in adolescent females. While these effects in adolescent males were abolished in mice lacking GHSR, adult male weight loss was maintained. Adolescent mice showed rebound hyperphagia on refeeding days without elevating cumulative food intake. The 5:2 diet elevated circulating free fatty acids by 30–57% independent of GHSR. However, inguinal white fat mass was elevated in adolescent males, by 51%, with interscapular brown fat mass elevated in males and females by 37%. The latter was accompanied by increased saturated fatty acid storage, in line with the inverse of thermogenic activity. The hepatic lipid profile was altered without impacting liver weight.

**Discussion:**

Thus, our data imply that while the 5:2 diet is an effective weight loss strategy in males, it has no impact on body weight in females. Despite elevating skeletal growth and lipolysis in line with the activity of the growth hormone-IGF-1 axis, this is countered by the bi-weekly ghrelin surges to increase fat mass in ghrelin sensitive depots. This combination of metabolic effects is undesirable, especially in the absence of obesity.

## Highlights

- The 5:2 diet induced compensatory rebound hyperphagia in male and female mice without increasing cumulative food intake.
- The 5:2 diet reduced weight gain in adolescent males and induced weight loss in adult males, without affecting body weight in female mice.
- The 5:2 diet increased tibial growth rate in adolescent mice, increasing marrow adiposity in males and decreasing marrow adiposity in females.
- The 5:2 diet elevated circulating free fatty acids and lipid storage in subcutaneous white adipose tissue and interscapular brown adipose tissue in male and female mice.
- These effects of the 5:2 diet on weight loss and skeletal growth in male mice and fat mass in both sexes were GHSR-dependent.

## Introduction

In the context of the ineffectiveness of many weight loss interventions for the alleviation of obesity, interest has grown in the potential health benefits of intermittent fasting (IF) (1–4). This feeding strategy takes several different forms, including time-restricted feeding (TRF), in which individuals restrict their food consumption to within a specified time window each day (usually <10 h), periodic fasting (PF) in which unrestricted feeding is interrupted by one-two days of complete abstinence per week, alternate-day fasting (ADF) comprised of 24 h periods of total or partial abstinence every other day, and modified alternate-day fasting (mADF) in which individuals fast for 2 non-consecutive days per week, with their remaining food intake otherwise unrestricted (4). The 5:2 diet is an ancient (Gospel of St Luke 18:12), but increasingly popular form of mADF (5–7), with a range of reported physiological benefits, such as weight loss, improved insulin sensitivity, enhanced cognitive performance and reduced disease risk (8, 9). However, given the range of potential risks, monitoring by trained practitioners is deemed essential (10, 11).

Despite its popularity, the exact mechanisms by which the 5:2 diet exerts these reported benefits remains poorly understood (12). The majority of the published pre-clinical studies to establish mechanistic pathways have been conducted in the context of diet-induced obesity (13, 14), with relatively few exploring the impact of this weight loss intervention in the absence of this complication (15, 16). One of the potential mediators linking dynamic alterations in dietary intake with a range of physiological outcomes is the gastric hormone ghrelin. Released in response to undernutrition and suppressed by refeeding (17), this acylated peptide binds to its receptor, GHSR, to integrate a range of physiological functions with nutritional status. These actions include, the stimulation of feeding behavior (18, 19), via GHSR in the hypothalamus (20, 21), growth hormone (GH) release (22) via GHSR in hypothalamus and pituitary (20, 23), fat accumulation (17, 24), via GHSR in white (25), and brown (26) adipocytes, including marrow adipocytes (25), suppression of gonadotrophin secretion (27), via hypothalamic GHSR and promotion of adult hippocampal neurogenesis via hippocampal GHSR (28, 29). Although GHSR is also expressed in bone (30), including osteoblasts (31), it is not thought to be present in the liver (32). Given that the impact of ghrelin on GH secretion and fat mass is dependent upon the dynamic pattern of exposure (33, 34), this hormone is well positioned to mediate any potential impact of the 5:2 diet on growth and metabolism.

In this study we have, therefore, explored the impact of the 5:2 diet in mice, characterizing its effects on body weight, skeletal growth and adiposity. These studies establish the effectiveness of this dietary intervention in male and female mice at two different ages (adolescent and adult) and explore its effectiveness in mice in which transcription of the receptor for ghrelin (GHSR), is blocked. The results obtained are both surprising and paradoxical.

## Materials and methods

### Animal studies

The animal procedures reported here (including those using genetically modified mice) were performed under the authority of the Animals (Scientific Procedures) Act, 1986 (United Kingdom) and were specifically approved by the Cardiff University Animal Welfare Ethical Review Body (AWERB). All subsequent reporting is in accordance with the ARRIVE guidelines.

WT (C57/Bl6J) mice and their homozygous loxTB-GHSR (GHSR-null) littermates (Studies 1 & 2) were obtained from heterozygous x heterozygous matings of breeding stock derived from mice imported from the vivarium at the University of Texas Southwestern (Dallas, TX, United States). Genotype identification was performed by PCR analysis of DNA extracted from ear punches, as previously described (35). WT mice (Study 3) were purchased from Charles River (Margate, United Kingdom).

All experimental animals were housed in the metabolic room of the BIOSV animal facility, Cardiff University under conditions of 12 h light/12 h dark (lights on at 06:00 h), with water available *ad libitum* and standard rodent chow (SDS RM3; Special Diet Services Ltd., United Kingdom; AFE 13.9% fat) supplied as described below:

### Study 1: the effect of the 5:2 diet on metabolic outcomes in adolescent mice

**Figure 1:**
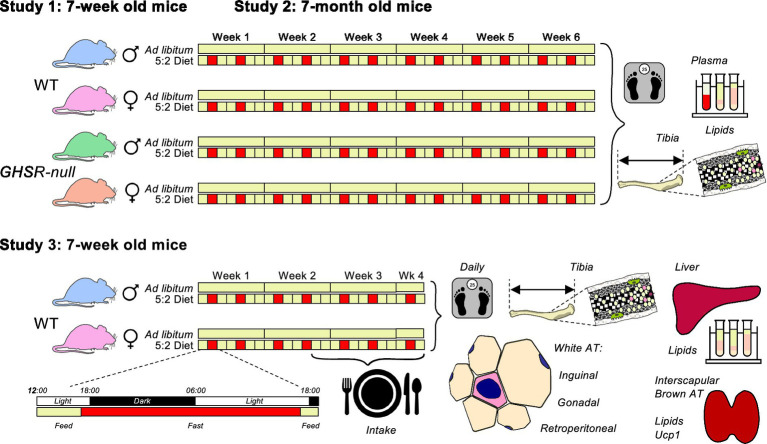
Experimental design to study the effects of the 5:2 diet on growth and metabolism (Studies 1, 2 & 3). 7-week old (Studies 1 & 3) and 7-month old (Study 2) male and female GHSR-null (Studies 1 & 2) and their wild-type (WT) littermates (Studies 1–3) received standard rodent chow either *ad libitum* or in a 5:2 diet, with the diet removed at 16:00 h on Mondays and Thursdays and replaced at 16:00 h on Tuesdays and Fridays. Body weight was monitored on Mondays, Wednesdays and Fridays (Studies 1 & 2), or daily (Study 3), with food intake monitored daily (days 12–24; Study 3). Indices of skeletal growth and metabolism were quantified in terminal tissue samples including in white and brown adipose tissue (AT).

Adolescent (7-week old) male [BW: 22.20 ± 0.43 g (*n* = 12)] and female [BW: 17.66 ± 0.40 g (*n* = 15)] and GHSR-null littermate mice [BW males: 20.97 ± 0.46 g (*n* = 14; *p* = 0.033 vs. WT males); BW females: 17.45 ± 0.43 g (*n* = 14; *p* = 0.363 vs. WT females)] were group housed (2–4 mice per cage) and received standard rodent chow either *ad libitum* (AL) or in a 5:2 pattern (*ad libitum*-feeding with diet removed for 24 h at 16:00 h on Mondays and Thursdays; *n* = 6–8 mice per group) for 6 weeks (Figure 1). Body weight was monitored between 09:00–10:00 h on Monday, Wednesday and Friday (i.e., on pre-fast, refed and fast days) and neurogenesis dependent behavior tested on days 42–45 [reported elsewhere (15)]. On day 46 animals were anaesthetized (Dolethal (200 mg/kg, i.p.); Vetoquinol UK Ltd., Towcester, United Kingdom), nose-anus length measured and blood samples collected by cardiac puncture before being killed by transcardial perfusion-fixation. Blood samples were treated as described below for subsequent quantification of hormone and lipid concentrations. Right tibiae were dissected, the length measured with a hand-held digital micrometer and processed as described below.

### Study 2: the effect of the 5:2 diet on metabolic outcomes in adult mice

Adult (7-month old) male [BW: 35.31 ± 1.28 g (*n* = 16)] and female [BW: 27.24 ± 0.79 g (*n* = 16)] WT and GHSR-null littermate mice [BW males: 31.29 ± 0.45 g (*n* = 15; *p* = 0.004 vs. WT males); BW females: 23.44 ± 0.61 g (*n* = 13; *p* = 0.001 vs. WT females)] were treated as in study 1 for the same period (Figure 1) with identical tissue samples collected under identical conditions.

### Study 3: the effect of the 5:2 diet on feeding and fat mass in adolescent mice

Adolescent (7-week old) male [BW: 24.16 ± 0.30 g (*n* = 12)] and female [BW: 17.71 ± 0.35 g (*n* = 12; *p* < 0.001 vs. males)] WT mice were group housed (3 mice per cage) and received standard rodent chow either *ad libitum* or in a 5:2 pattern as in Study 1 for 24 days (Figure 1). On day 11, each mouse was individually housed in a metabolic cage (Tecniplast metabowl Cat # 3700 M061; Tecniplast UK Ltd), the feeding patterns continuing uninterrupted and with food intake monitored daily (between 09:00–10:00 h). Body weight was measured daily throughout (at 09:00–10:00 h). On day 24 mice were anaesthetized as in study 1, body length measured, and decapitated. Trunk blood was collected and treated as described below for subsequent quantification of hormone concentrations. Inguinal, gonadal and retroperitoneal white adipose tissue (WAT) depots were dissected, along with interscapular brown AT (BAT), liver and kidney, and weighed, subsamples being retained for further analysis (see below). Tibiae were also dissected, measured and processed as in study 1.

### Tissue analysis

#### Quantification of tibial growth rates and adiposity

Tibial epiphyseal plate width (EPW) and marrow adiposity were quantified as previously described (34, 36). In brief, tibiae were post-fixed in 4% paraformaldehyde, decalcified in 10% EDTA for 3 weeks and paraffin-embedded. EPW was measured on 7 μm Masson’s trichrome-stained sections under light microscopy (Leica DMLB fitted with a Leica DFC300FX camera) using Leica Q-win software (v3). Marrow adiposity (%-field, adipocyte number and size) was quantified on digital images of mid-diaphyseal bone marrow from the same sections using National Institutes of Health (NIH) Image J for Mac.^1^

Inguinal and gonadal WAT adipocyte size were determined as previously described (25). In brief, WAT samples were post-fixed in 4% paraformaldehyde and paraffin-embedded, 7 μm microtome sections being stained with Masson’s trichrome. Digital images were obtained under light microscopy as above, with adipocyte size being measured only on cells with a visible peripheral elliptical nucleus. Similarly stained BAT sections were visualized in the same way, with 10 μm cryostat liver sections stained with haematoxylin and eosin. Uncoupling protein-1 (*Ucp1*) mRNA expression in BAT was quantified by qPCR as previously described (37). In brief, total RNA was extracted from lysed tissue using an RNeasy Lipid Tissue Mini Kit (Qiagen), reverse transcribed using a Precision nanoScript 2 RT Kit. *Ucp-1* and *β-actin* cDNA were quantified in 1:10-diluted samples using oligonucleotide primers (Integrated DNA Technologies (*Ucp1*) and Primerdesign (*β-actin*); sequences as used previously (37)), with values from individual samples normalized to *β-actin*.

Circulating free fatty acids (FFAs), lipid accumulation and FA profiles in plasma, BAT and liver samples were determined by gas chromatography-flame ionization detection (GC-FID) as previously described (38). Total lipids were extracted according to the method of Folch et al. (39). FFAs were separated from the total lipid pool using one-dimensional thin-layer chromatography (TLC) on silica gel G plates (10 × 10 cm, Supelco, Sigma-Aldirch UK). The solvent mixture used was 80:20:1 (v/v/v) hexane/diethyl ether/acetic acid. Following separation, the plates were dried and sprayed with a 0.05% solution of 8-anilino-4-naphthosulphonic acid in methanol (w/v). FFA bands were visualized under UV light, scraped off the plates along with the silica gel and analyzed as described below. Aliquots of extracted lipids and the separated FFAs were used for fatty acid methyl ester (FAME) preparation. FAMEs were prepared by transmethylation with 2.5% H_2_SO_4_ in dry methanol / toluene (2:1, v/v) at 70 °C for 2 h. A known amount of nervonic acid (C24:1n9) was added as an internal standard, so that subsequent quantification of peaks (and, consequently, lipids) could be performed. FAMEs were extracted with HPLC-grade hexane. A Clarus 500 gas chromatograph with a flame ionizing detector (FID; Perkin-Elmer 8,500, Norwalk, CT, United States) and fitted with a 30 m 0.25 mm i.d. capillary column (Elite 225, Perkin Elmer) was used for separation and analysis of FAs. The oven temperature was programmed as follows: 170 °C for 3 min, programmed to 220 °C at 4 °C/min, hold for 15 min. FAMEs were identified routinely by comparing retention times of peaks with those of G411 standards (Nu-Chek Prep. Inc., Elysian, MN, United States). Perkin-Elmer Total Chrom Navigator software was used for data acquisition.

#### Hormone quantification

Blood samples were collected into Lavender Vacutainer EDTA blood tubes (#VT-6450) and treated with AEBSF (2 mg/mL, Cat No. A8456 Sigma) before being centrifuged and the plasma collected and stored at −80 °C prior to hormone quantification. Plasma IGF-1 concentrations were determined by ELISA (R&D Systems Mouse/Rat IGF-1 DuoSet DY791) according to manufacturer’s instructions. Plasma corticosterone concentrations were determined by ELISA (ENZO Life Sciences, ADI-900-097) following the manufacturers guidelines, as previously described (15). ELISA plates were read on a POLARstar Omega (BMG Labtech) plate reader.

#### Statistics

All data are presented as mean ± SEM. The effect of the 5:2 diet in each sex/genotype group was assessed with Students t-test, using MS Excel for Mac version 16.86, with *p* < 0.05 considered statistically significant.

## Results

### Study 1: the effect of the 5:2 diet on metabolic outcomes in adolescent mice

**Figure 2:**
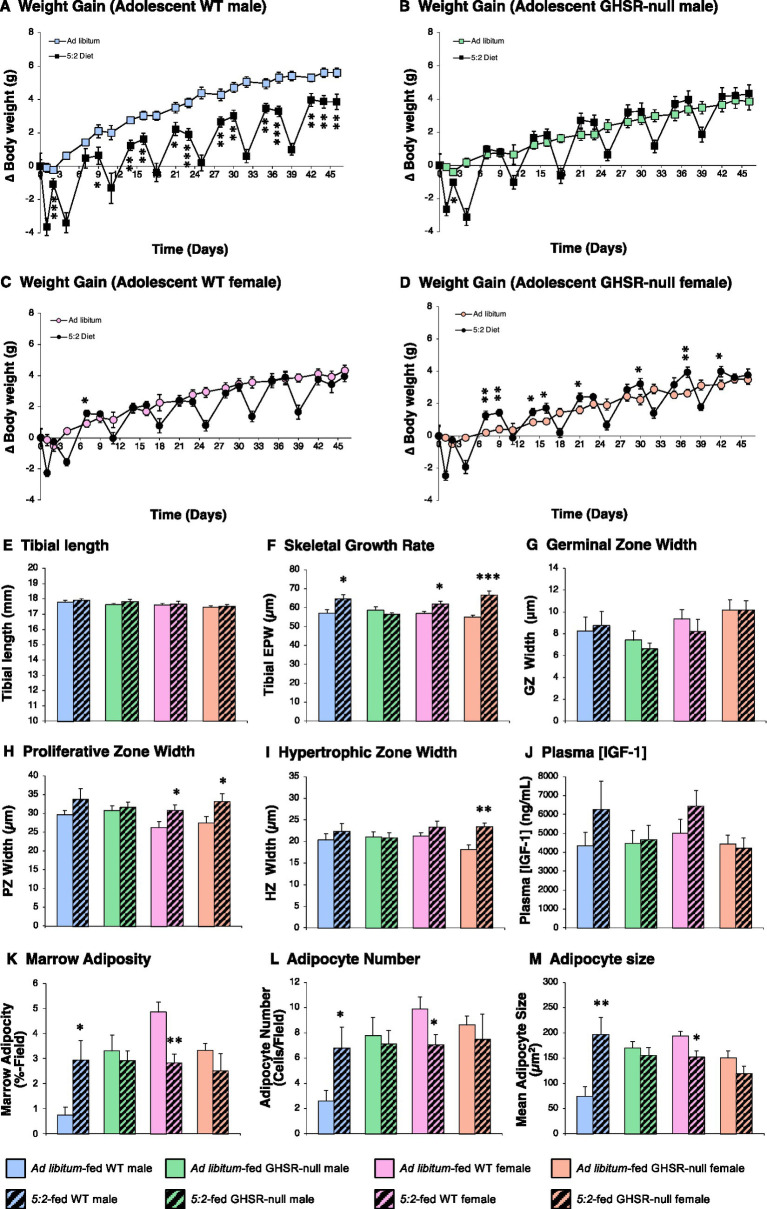
The 5:2 diet reduces weight gain in adolescent male mice by a GHSR-dependent mechanism (Study 1). The effect of 6 weeks of *ad libitum* (AL)-feeding (plain symbols/bars) or 5:2-feeding (black symbols/hatched bars) on body weight gain **(A–D)**, and tibial length **(E)**, epiphyseal plate width (EPW; **F**), germinal (GZ; **G**), proliferative (PZ; **H**) and hypertrophic (HZ; **I**) zone widths, tibial marrow adiposity **(K)**, marrow adipocyte number **(L)** and size **(M)** and plasma IGF-1 concentration **(J)** in adolescent (7-week old) male (**A**; blue symbols/bars) and female (**C**; pink symbols/bars) WT and GHSR-null male (**B**; green symbols/bars) and female (orange symbols/bars) mice. Data shown are mean ± SEM [*n* = 6 (WT males (both diets)], 7 [GHSR-null males (both diets), AL-fed WT females and GHSR-null females (both diets)] and 8 (5:2-fed WT females), with statistical comparisons of the impact of the 5:2 diet within each sex/genotype group performed by unpaired Students t-test (*p < 0.05; **p < 0.01; ***p < 0.001 vs. AL-fed).

To determine the impact of the 5:2 diet in mice during the steepest period of their growth phase, 7-week old male and female WT mice, together with GHSR-null littermates were AL-fed or maintained on a 5:2 diet for 6 weeks (Figure 1). The 5:2 diet produced a 31% reduction in body weight gain in WT males (Figure 2A; *p* = 0.004), the first refeeding day on which this became apparent being day 9. However, the 5:2 diet failed to induce a parallel reduction in either nose-anus length (data not shown) or tibial length (Figure 2E). Indeed, quantification of tibial epiphyseal plate width (EPW), the most accurate index of the rate of growth at the time of death, demonstrated that skeletal growth rate was increased by 13% in 5:2-fed males (Figure 2F; *p* = 0.014). This increased growth rate appeared to be due to a combination of effects on the proliferative and hypertrophic zones (Figures 2H,I), though neither were significantly different alone. While the overall profile of circulating IGF-1 paralleled these changes in skeletal growth rate (Figure 2J), none of the means were significantly different due to the high degree of variation. In addition, the 5:2 diet induced a 3-fold increase in tibial marrow adiposity (Figure 2K; *p* = 0.018), reflected in a doubling of adipocyte number (Figure 2L; *p* = 0.033) and size (Figure 2M; *p* = 0.008).

Unlike in males, the 5:2 diet had no effect on weight gain in adolescent WT females (Figure 2C), except a transient overshoot on day 7. Although neither body length nor tibial length were affected in females (Figure 2E), tibial EPW was elevated by 9% (Figure 2F; *p* = 0.020) primarily as a result of a widening of the proliferative zone (Figure 2H; *p* = 0.020), with neither germinal nor hypertrophic zone widths being significantly different (Figures 2G,I). However, in stark contrast to the impact in males, marrow adiposity was almost halved by the 5:2 diet in WT females (Figure 2K; *p* = 0.001), due to a 29% reduction in adipocyte number (Figure 2L; *p* = 0.019) and a 22% reduction in adipocyte size (Figure 2M; *p* = 0.010).

These effects of the 5:2 diet on weight gain, acceleration of tibial growth and elevation in marrow adiposity in male mice were all abolished in the absence of ghrelin signaling (Figure 2). In contrast, the overshoot in weight gain that occurred following re-feeding in WT females was exaggerated in GHSR-null females and the accelerated tibial growth rate was augmented in 5:2 diet-fed GHSR-null females (Figure 2F; *p* = 0.001). The latter was due to increases in proliferative and hypertrophic zone widths (Figures 2H,I; *p* = 0.028 and 0.001 respectively). Interestingly, the reduction in marrow adiposity in WT females was not significant in the absence of GHSR (Figure 2K; *p* = 0.150).

Thus, in males the 5:2 diet partitions resources to protect skeletal growth and marrow fat in the context of reduced weight gain via changes in ghrelin activity. In contrast, females show no slowing of weight gain with only the reduction in marrow adiposity being GHSR-dependent.

### Study 2: the effect of the 5:2 diet on metabolic outcomes in adult mice

**Figure 3:**
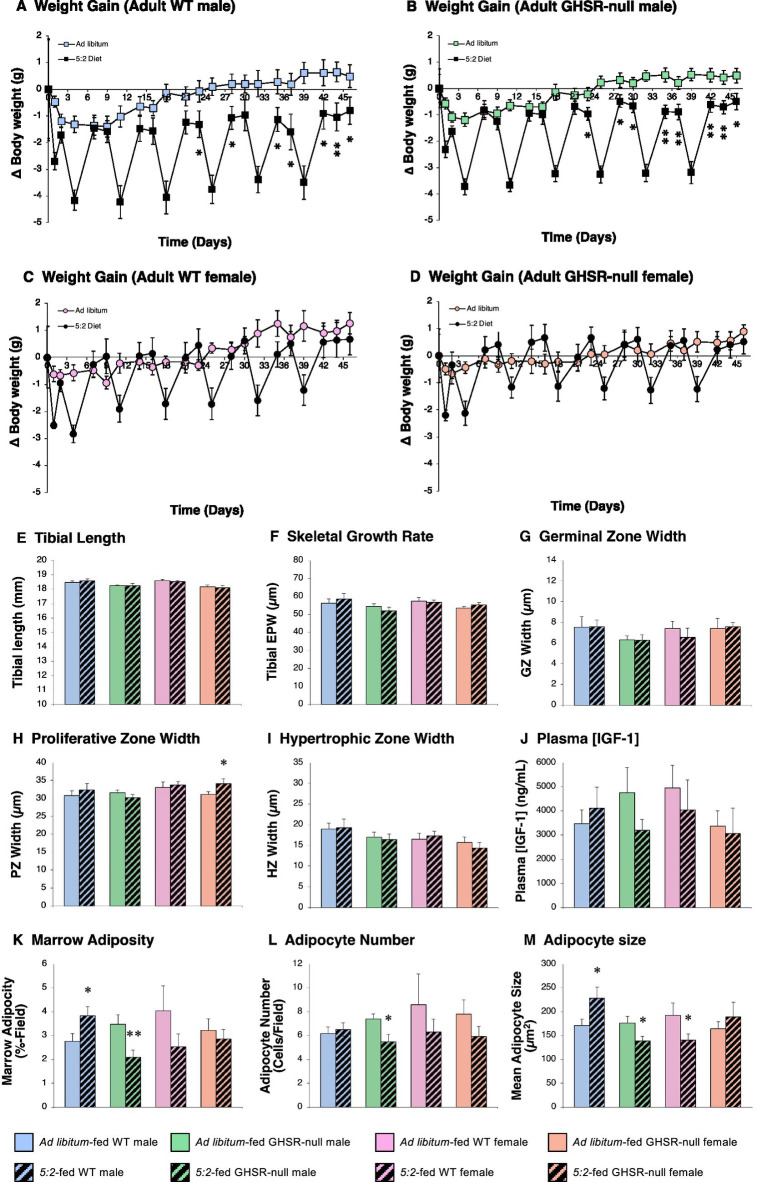
The 5:2 diet reduces weight loss in adult male mice by a GHSR-independent mechanism (Study 2). The effect of 6 weeks of (*AL*)-feeding (plain symbols/bars) or 5:2-feeding (black symbols/hatched bars) on body weight gain **(A–D)**, and tibial length **(E)**, epiphyseal plate width (EPW; **F**), germinal (GZ; **G**), proliferative (PZ; **H**) and hypertrophic (HZ; **I**) zone widths, tibial marrow adiposity **(K)**, marrow adipocyte number **(L)** and size **(M)** and plasma IGF-1 concentration **(J)** in adult (7-month old) male (**A**; blue symbols/bars) and female (**C**; pink symbols/bars) WT and GHSR-null male (**B**; green symbols/bars) and female (orange symbols/bars) mice. Data shown are mean ± SEM [*n* = 6; WT males (both diets)], 7 [GHSR-null males (both diets), AL-fed WT females and GHSR-null females (both diets)] and 8 (5:2-fed WT females), with statistical comparisons of the impact of the 5:2 diet within each sex/genotype group performed by unpaired Students t-test (*p < 0.05; **p < 0.01 vs. AL-fed).

To determine whether these effects are replicated in older animals that are past the steep phase of the growth curve, study 1 was repeated in adult (7-month old) mice (Figure 1). The 5:2 diet induced a sustained weight loss in WT males (Figure 3A), the first refeeding day on which this was observable being day 23 (*p* = 0.037). Unsurprisingly, given the age of these mice, this reduction in body weight was not accompanied by any observable change in tibial length or growth rate (Figures 3E,F), any of its component elements (Figures 3G–I), or circulating IGF-1 (Figure 3J). However, tibial marrow adiposity was elevated by 39% in 5:2-fed WT males (Figure 3K; *p* = 0.030), as previously observed in adolescent WT males.

As seen in adolescent females, the 5:2 diet had no effect on weight gain (Figure 3C) or any of the indices of skeletal growth in adult females (Figures 3E–I). In addition, although mean marrow adiposity in adult WT females on the 5:2 diet was only 63% of that in AL-fed WT females, this was not significantly different (Figure 3K; *p* = 0.112), adipocyte size being reduced by 27% (Figure 3M; *p* = 0.049).

In contrast with younger mice, the effect of the 5:2 diet on weight gain in male mice was maintained in the absence of ghrelin signaling (Figure 3B), the change in body weight in GHSR-null males at day 45 being −0.49 ± 0.31 g (*p* = 0.016 vs. AL-fed GHSR-null males). Loss of GHSR also reversed the positive impact of the 5:2 diet on marrow adiposity, marrow adiposity being reduced by 40% in 5:2-fed GHSR-null males (Figure 3K; *p* = 0.009). This was due to a combination of a 26% reduction in adipocyte number (Figure 3L; *p* = 0.014) and a 21% reduction in adipocyte size (Figure 3M; *p* = 0.027). Loss of GHSR had little impact on the effect of the 5:2 diet in female mice (Figures 3D–M), the reduction in marrow adipocyte size being reversed (Figure 3M).

Thus, the impact of the 5:2 diet on body weight and marrow adiposity in adult mice is also male-specific, with the increase in marrow adiposity being dependent upon GHSR.

### Study 3: the effect of the 5:2 diet on feeding and fat mass in adolescent mice

To determine whether the changes seen above in adolescent mice are accompanied by alterations in energy intake and wider metabolic disturbances, a second cohort of 7-week old male and female WT mice were maintained on a 5:2 diet or AL-fed for 3 weeks (Figure 1). After an initial period of group-housing (3 mice per cage) mice were transferred on day 11 to individual cages for monitoring of daily food intake.

**Figure 4:**
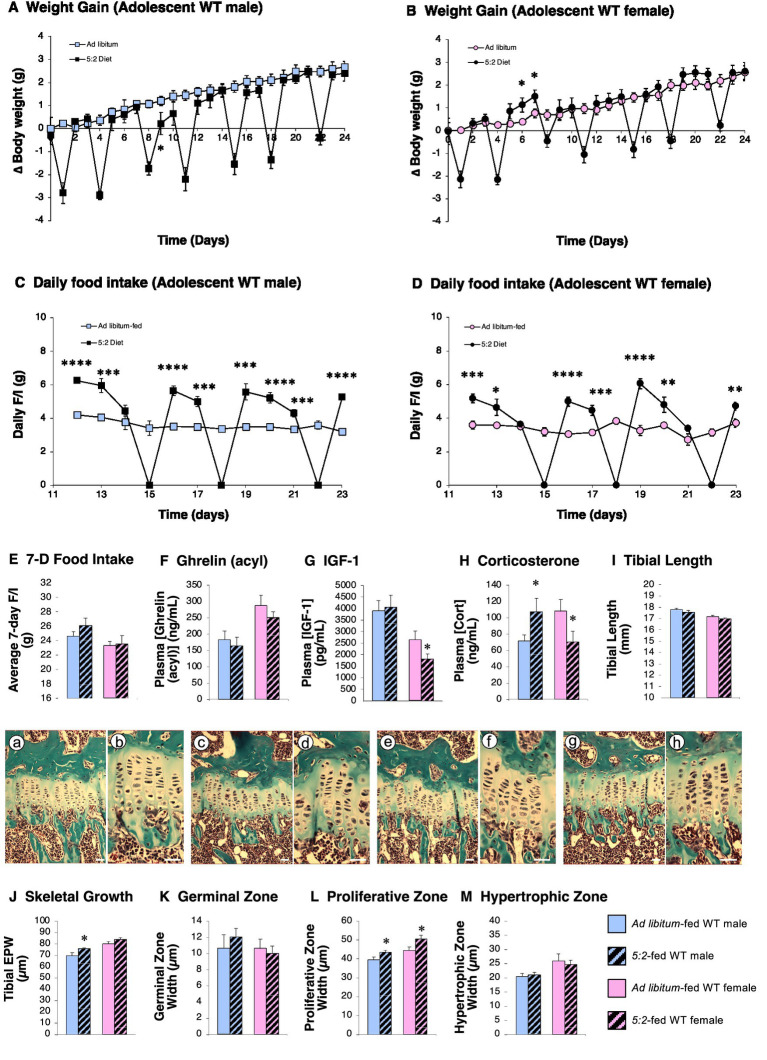
The 5:2 diet induces hyperphagia in male and female mice (Study 3). The effect of 3 weeks of *ad libitum* (AL)-feeding (plain symbols/bars) or 5:2-feeding (black symbols/hatched bars) on body weight gain **(A,B)**, daily food intake (F/I) **(C,D)**, mean-7-day food intake **(E)**, circulating acyl ghrelin (**F**), IGF-1 **(G)**, corticosterone **(H)**, tibial length **(I)**, epiphyseal plate width (EPW; **J**), germinal (GZ; **K**), proliferative (PZ; **L**) and hypertrophic (HZ; **M**) zone widths in adolescent (7-week old) male and female WT mice. Inset pictures show representative low and high magnification images of EPWs from AL-fed males (a,b) 5:2-fed males (c,d), AL-fed females (e,f) and 5:2-fed females (g,h). Data shown are mean ± SEM [*n* = 9 (all groups)], with statistical comparisons of the impact of the 5:2 diet within each sex performed by unpaired Students t-test (*p < 0.05; **p < 0.01; ***p < 0.001; ****p < 0.0001 vs. AL-fed).

Male WT mice receiving the 5:2 diet showed the same initial reduction in body weight as those in study 1, with body weight gain reduced by 82% on day 9 (Figure 4A; *p* = 0.040). However, once transferred to individual housing, the growth rate of AL-fed WT males slowed, resulting in no significant difference in body weight gain after 21 days. As in study 1, female WT mice maintained on the 5:2 diet showed an initial period of weight overshoot, weight gain on day 6 being elevated 3-fold (Figure 4B; *p* = 0.016). Thereafter 5:2-fed WT females showed the same weight gain as AL-fed mice. Both male and female mice showed significant hyperphagia on non-fast days (Figures 4C,D). Although in females the degree of hyperphagia exactly matched the calories denied on the fast days (Figure 4E; 101.3%; *p* = 0.408), mean 7-day food intake over the entire monitoring period in 5:2-fed males was 106% of that in AL-fed males, though these values were not significantly different (Figure 4E; *p* = 0.124).

Neither pituitary, adrenal, nor kidney weights (Table 1) were affected by the 5:2 diet in either sex. Analysis of terminal plasma samples collected on day 24 (the first refeeding day of the 5:2 cycle) revealed that while the 5:2 diet had no impact on circulating ghrelin (acylated; Figure 4F), or on IGF-1 concentration in males (Figure 4G), circulating IGF-1 was reduced by 31% in 5:2-fed females (Figure 4G; *p* = 0.045). Interestingly, the 5:2 diet also reduced circulating corticosterone in females by 35% (Figure 4H; *p* = 0.035), but elevated it by 50% in males (Figure 4H; *p* = 0.036).

**Table 1:** Changes in post-mortem organ weights in 5:2-diet-fed mice.

	AL-fed male (*n* = 9)	5:2-fed male (*n* = 9)	AL-fed female (*n* = 9)	5:2-fed female (*n* = 9)
Pituitary weight (mg)	0.94 ± 0.12	1.09 ± 0.12	1.42 ± 0.30	1.19 ± 0.11
Adrenal weight (mg)	2.48 ± 0.15	2.49 ± 0.20	3.81 ± 0.17	3.63 ± 0.25
Kidney weight (%-BW)	0.594 ± 0.008	0.583 ± 0.010	0.586 ± 0.018	0.585 ± 0.009
Liver weight (g)	1.24 ± 0.13	1.33 ± 0.05	1.01 ± 0.06	1.20 ± 0.06
Total hepatic lipid content (mg FA/1 g)	22.80 ± 0.88	23.28 ± 1.00	26.80 ± 2.01	31.88 ± 2.88

As in study 1, although tibial length was not significantly affected (Figure 4I), the rate of skeletal growth was increased by 9% in males (Figure 4J; *p* = 0.027) in 5:2-fed mice, this not reaching statistical significance in females (Figure 4J; *p* = 0.074). These changes were the result of 10 and 13% increases in the width of the proliferative zone in 5:2-fed males and females, respectively, (Figure 4L; *p* = 0.016 & 0.023), with neither germinal nor hypertrophic zone widths being affected (Figures 4K,M).

**Figure 5:**
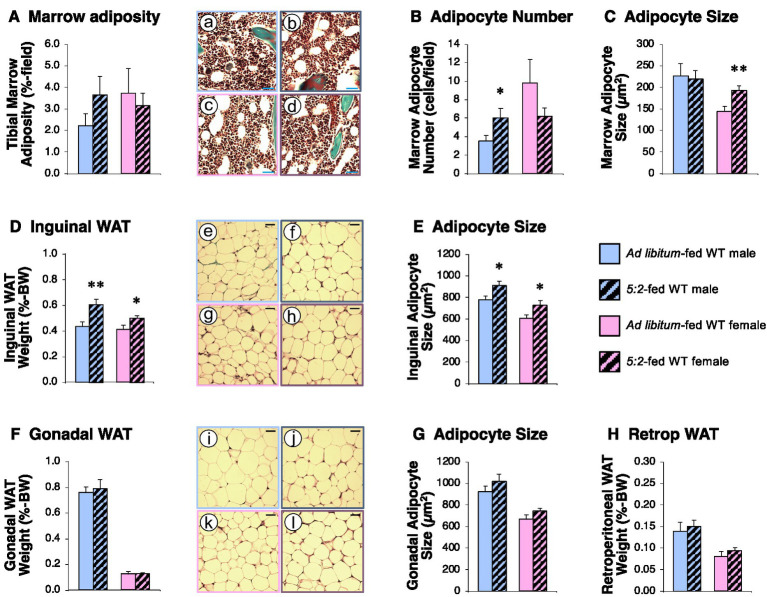
The 5:2 diet induces depot-specific elevation in adiposity in male and female mice (Study 3). The effect of 3 weeks of *ad libitum* (AL)-feeding (plain bars) or 5:2-feeding (hatched bars) on tibial marrow adiposity **(A)**, adipocyte number **(B)** and adipocyte size **(C)**, inguinal white adipose tissue (WAT) weight **(D)**, and adipocyte size **(E)** gonadal (epididymal or ovarian) WAT weight **(F)** and adipocyte size **(G)** and retroperitoneal (Retrop) WAT weight **(H)** in adolescent (7-week old) male (blue bars) and female (pink bars) WT mice. Inset pictures show representative images of marrow adiposity (a–d) inguinal WAT (e–h) and gonadal WAT (i–l) in AL-fed males (a,e,i), 5:2-fed males (b,f,j), AL-fed females (c,g,k) and 5:2-fed females (d,h,l; scale bars: 20 μm). Data shown are mean ± SEM [*n* = 9 (all groups)], with statistical comparisons of the impact of the 5:2 diet within each sex performed by unpaired Students t-test (*p < 0.05; **p < 0.01 vs. AL-fed same sex).

The 5:2 diet elicited broadly similar changes in marrow adiposity to those seen in study 1. Mean marrow adiposity in 5:2-fed males was 164% of that in AL-fed males (*p* = 0.095; Figure 5A) as a result of a 72% increase in adipocyte number (*p* = 0.029; Figure 5B). In contrast, while adipocyte size was increased by 34% in 5:2-fed females (*p* = 0.005; Figure 5C), overall marrow adiposity was unaltered (Figure 5A). In addition, neither gonadal (epididymal or ovarian; Figure 5F) nor retroperitoneal (Figure 5H) WAT depot weights were influenced by the 5:2 diet in male or female mice. In contrast, the 5:2 diet elevated proportionate inguinal WAT weight by 39 and 22% in male and female mice, respectively, (Figure 5D; *p* = 0.004 and 0.024). This was reflected in 17 and 20% increases in inguinal adipocyte size in males and females (Figure 5E; *p* = 0.016 and 0.019), gonadal adipocyte size being unaltered (Figure 5G).

**Figure 6:**
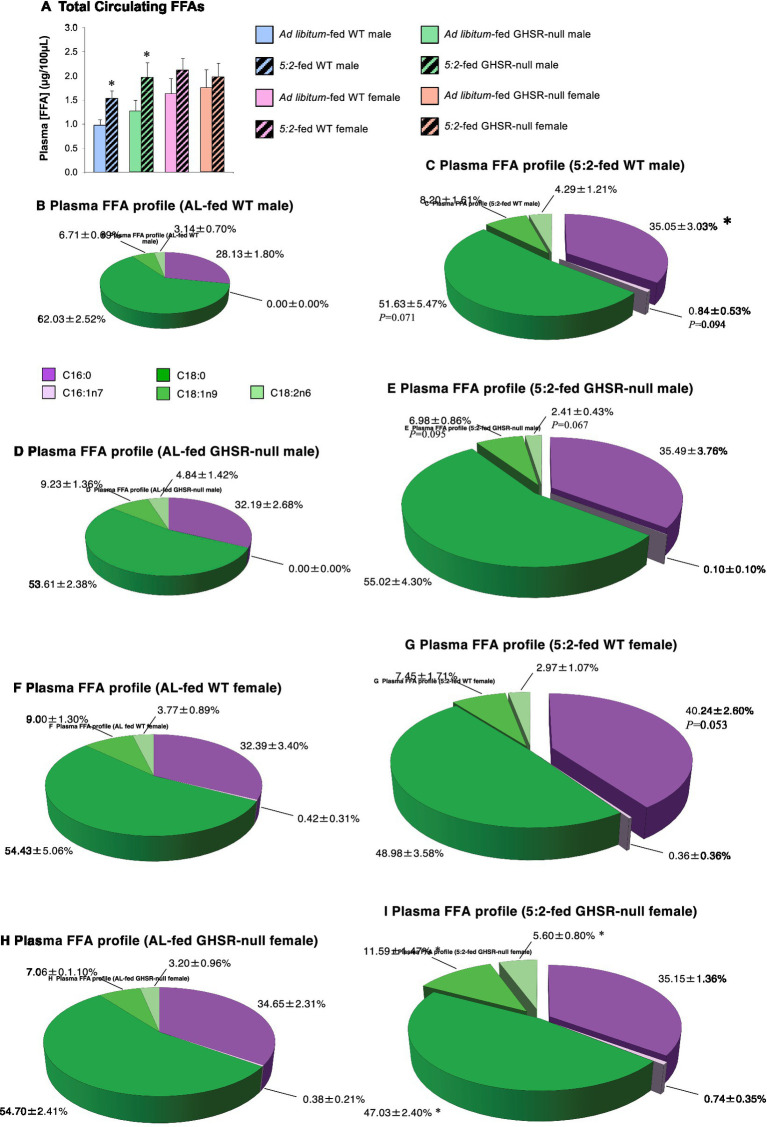
The 5:2 diet stimulates lipolysis in male mice independent of GHSR signaling (Study 1). The effect of 6 weeks of *ad libitum* (AL)-feeding (plain symbols/bars) or 5:2-feeding (black symbols/hatched bars) on total plasma FFAs **(A)**, and the profile of major plasma FFAs **(B–I)** in adolescent (7-week old) male (blue symbols/bars and **B,C**) and female (pink symbols/bars and **F,G**) WT and GHSR-null male (green symbols/bars and **D,E**) and female (orange symbols/bars and **H,I**) mice. Pie charts **(B–I)** show the proportion (%) of individual FA species, the diameter of the unexploded pie reflecting mean total FFA concentration. Reduced, unchanged and increased FA values in 5:2-fed mice (vs. AL-fed mice; **D,F**) are unmoved, partially exploded and fully exploded, respectively. Data shown are mean ± SEM [*n* = 5; AL-fed WT males and 5:2-fed WT females(both diets)], 6 {5:2-fed WT males, GHSR-null males (both diets) and 7 [AL-fed WT females and GHSR-null females (both diets)]}, with statistical comparisons of the impact of the 5:2 diet within each sex/genotype group performed by unpaired Students t-test [*p < 0.05 vs. AL-fed (same sex/genotype)].

Given these changes in WAT mass, we investigated whether the 5:2 diet induced changes in circulating FFAs in plasma samples from study 1. After 6 weeks on the 5:2 diet, total plasma FFA concentration was increased in WT males by 57% (*p* = 0.023; Figure 6A). This was accompanied by a shift in the FFA profile in favor of palmitic acid (C16:0; 24% elevation; *p* = 0.048; Figure 6C), with mean stearic acid (C18:0) concentration being 83% of that in AL-fed WT males (*p* = 0.071; Figures 6B,C). These effects were less prominent in females, mean total FFA concentration in 5:2-fed WT females being 130% of that in AL-fed females (*p* = 0.135; Figure 6A) and there being no significant changes in the proportion of individual FFAs (Figures 6F,G). This lipolytic impact of the 5:2 diet was independent of GHSR-signaling, being largely replicated in GHSR-null males (total FFA concentration elevated by 55%; *p* = 0.045; Figures 6A,D,E) and females (Figures 6A,H,I).

**Figure 7:**
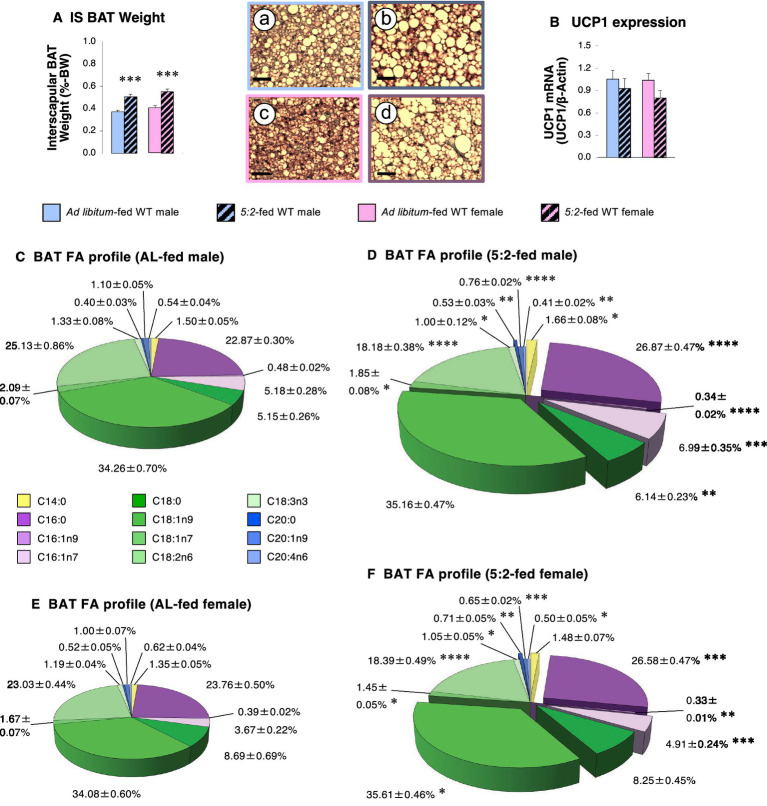
The 5:2 diet promotes lipid storage in brown adipose tissue of male and female mice (Study 3). The effect of 3 weeks of *ad libitum* (AL)-feeding (plain bars) or 5:2-feeding (hatched bars) on the proportionate weight **(A)**, UCP1 mRNA expression (**B**; fold change against *β*-actin) and the fatty acid (FA) profile **(C–F)** of interscapular brown adipose tissue (BAT) in adolescent (7-week old) male (blue bars; **A,B**) and female (pink bars; **A,B**) WT mice. Pie charts show the proportion (%) of individual FA species, the diameter of the unexploded pie reflecting mean BAT mass. Reduced, unchanged and increased FA values in 5:2-fed mice (vs. AL-fed mice; **D,F**) are unmoved, partially exploded and fully exploded, respectively. Inset pictures show representative images of interscapular BAT from AL-fed males **(A)**, 5:2-fed males **(B)**, AL-fed females **(C)** and 5:2-fed females **(D)** (scale bars: 20 μm). Data shown are mean ± SEM [*n* = 9 (all groups)], with statistical comparisons of the impact of the 5:2 diet within each sex performed by unpaired Students t-test [*p < 0.05; **p < 0.01; ***p < 0.001; ****p < 0.0001 vs. AL-fed (same sex)].

Given this combination of elevated lipid availability and depot-specific increases in WAT mass, we investigated whether there were any broader changes in lipid storage. The 5:2 diet elevated interscapular BAT weight by 37% in both male and female mice (Figure 7A; *p* = 0.0001 for both sexes) and this was accompanied by a marked change in histological appearance, BAT sections from 5:2-fed mice showing a marked increase in the density of lipid droplets (Figure 7 inset pictures). Mean normalized *Ucp1* mRNA expression in 5:2-fed males and females was 88 and 77% of that in AL-fed mice (Figure 7B; *p* = 0.189 & *p* = 0.0503 vs. same sex, respectively). GC-FID lipid profiling in BAT samples revealed that the 5:2 diet induced an identical change in the lipid profile in male and female mice. 11–35% increases in the relative amounts of saturated FAs [myristic acid (C14:0), palmitic acid, stearic acid and arachidic acid (C20:0)] occurred in BAT of both sexes (Figures 7C–F; *p* < 0.05), with 12–35% reductions in monounsaturated fatty acids [MUFAs; *cis*-7-hexadecenoic acid (C16:1n9), *cis*-vaccenic acid (18:1n7) and eicosenoic acid (C20:1n9)] and polyunsaturated fatty acids [PUFAs; linoleic acid (C18:2n6), α-linolenic acid (C18:3n3) and arachidonic acid (C20:4n6); Figures 7C–F; *p* < 0.05].

**Figure 8:**
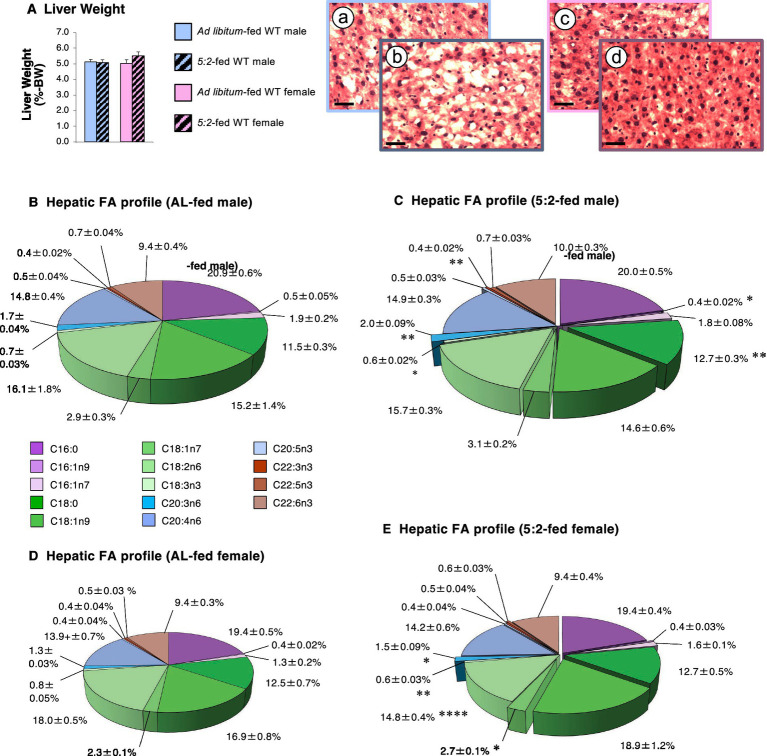
The 5:2 diet elevates hepatic lipid storage in male and female mice (Study 3). The effect of 3 weeks of *ad libitum* (AL)-feeding (plain bars) or 5:2-feeding (hatched bars) on proportionate hepatic weight **(A)** and hepatic fatty acid (FA) profile **(B–E)** in adolescent (7-week old) male (blue bars **A**) and female (pink bars **A**) WT mice. Pie charts show the proportion (%) of individual FA species in AL-fed males and females **(B,D)** and 5:2-fed males and females **(C,E)**, the diameter of the unexploded pie reflecting mean liver weight. Reduced, unchanged and increased FA values in 5:2-fed mice (vs. AL-fed mice; **C,E**) are unmoved, partially exploded and fully exploded, respectively. Inset pictures show representative images of cryostat sections of liver from AL-fed males **(A)**, 5:2-fed males **(B)**, AL-fed females **(C)** and 5:2-fed females **(D)** (scale bar: 20 μm). Data shown are mean ± SEM [*n* = 9 (all groups)], with statistical comparisons of the impact of the 5:2 diet within each sex performed by unpaired Students t-test [*p < 0.05; **p < 0.01; ****p < 0.0001 vs. AL-fed (same sex)].

Given these changes in lipid storage in BAT, we used the same approach in liver samples to determine whether increased lipid storage was more widespread. Despite the absence of a significant change in actual (Table 1) or proportionate liver weight (Figure 8A), histological visualization of stained liver sections (Figures 8A–D) suggested that while lipid content may be higher in males, the 5:2 diet may only exert a minimal influence on hepatic lipid storage. Lipid profiling revealed that mean total lipid content in 5:2-fed females was 119% of that in AL-fed females (*p* = 0.084) and unaffected in males (Table 1). Although the relative amounts of palmitic, arachidonic and docosahexaenoic (C22:6n3) acids were unaffected by the 5:2 diet in both sexes (Figures 8B–E), the proportion of stearic acid was elevated by 10% in males (*p* = 0.007), but unchanged in females. The relative proportions of some MUFAs and PUFAs were elevated in both sexes [dihomo-γ-linolenic acid (C20:3n6) was elevated by 17% (*p* < 0.05)], elevated only in males [eicosapentaenoic acid (C20:5n3) was 120% of that in AL-fed males (*p* = 0.050); docosatrienoic acid (C22:3n3) was elevated by 19% (*p* = 0.005)], or elevated only in females [palmitoleic acid (C16:1n7) was 120% of that in AL-fed females (*p* = 0.084), oleic acid (C18:1n9) was 112% of that in AL-fed females (*p* = 0.092); *cis*-vaccenic acid was increased by 19% (*p* = 0.010); docosapentaenoic acid (C22:5n3) was 113% of that in AL-fed females (*p* = 0.057)]. In addition, some MUFAs and PUFAs were reduced in both sexes [α-linolenic acid was reduced by 14 and 26% in males and females, respectively, (*p* < 0.05)], only in males [*cis*-7-hexadecenoic acid was reduced by 21% (*p* = 0.049)], or only in females [linoleic acid was reduced by 19% (*p* = 0.0001)]. Thus, the 5:2 diet elicits sex-specific changes in the FA profile of total lipids in other metabolically active tissues.

## Discussion

The 5:2 diet is a weekly intermittent fasting strategy in which participants consume little to no calories on two non-consecutive days, eating normally for the remaining 5 days. Despite its popularity as a weight loss strategy, we were surprised to find that in non-obese mice maintained on a standard rodent chow the effectiveness of the 5:2 diet was both sex-dependent and accompanied by paradoxical physiological endpoints, many of which were wholly dependent upon or modulated by the activity of ghrelin at its receptor, GHSR.

### Impact of the 5:2 diet on body weight

Several previous studies have reported that intermittent fasting results in reduced weight gain (16, 40), so we were surprised to find that in mice the 5:2 diet only elicited weight loss or reduced weight gain in males, female body weight being entirely unaffected. Indeed, closer examination of the published human studies confirms that many were performed exclusively in males (16, 40). A number of potential mechanisms could give rise to this sexually-dimorphic effect. The first, is the possibility of a sex-specific difference in overall food consumption. This appears not to be the case, our study revealing that while both genders display hyperphagia on the re-feeding days, this exactly matched the consumption denied during the fast days rather than producing over-compensatory hyperphagia. Similar findings have recently been reported for female mice given a once-weekly 24 h or 48 h fast (41). The basis for this weight loss in males must therefore be found in sex-specific differences in underlying anatomical and metabolic responsiveness.

### Contribution of skeletal growth

One contributor to body weight gain is skeletal growth. While the duration of our studies were insufficient to yield detectable changes in bone length, our quantification of tibial EPW indicates that the rate of tibial growth was enhanced in both sexes by the 5:2 diet, even in older animals past the steepest section of the growth curve. Interestingly, this growth acceleration was most clearly reflected in the proliferative zone of the growth plate where growth hormone (GH) and IGF-1 promote clonal expansion of the chondrocytes. The fact that this growth acceleration and the accompanying weight loss were only reversed by the absence of GHSR in adolescent mice when the growth curve is the steepest, suggests that, at this younger age at least, the 5:2 diet activates the ghrelin-growth hormone (GH) axis. Indeed, we have recently reported that meal-feeding enhances growth hormone (GH) rhythmicity in males (42), implying that intermittent fasting may protect or enhance GH-dependent growth. Given the robust episodic nature of GH secretion in males, it would be informative to characterize GH secretory profiles, but this is technically challenging in mice. However, terminal circulating IGF-1 levels, often used as a proxy for GH axis activity, though not statistically significant, did show a parallel overall diet-dependent pattern. Our data contrast with that presented by Varady *et al.* (11), who reported that mADF reduced circulating IGF-1 in mice. These different outcomes may reflect study duration, the absolute pattern of intermittent fasting or the timing of sample collection relative to the fasting-refeeding cycle.

Interesting as these effects are, the sex-independent acceleration of skeletal growth is unlikely to contribute to the male-specific decline in body weight.

### Contribution of fat mass

An alternative mechanism could be a decline in fat mass. However, this also does not appear to be the case as the 5:2 diet was seen to elevate inguinal WAT mass (a deep subcutaneous depot) in both sexes accompanied by a parallel increase in adipocyte size. Our data contrasts previous reports that ADF reduces adipocyte size in male mice without affecting inguinal and epididymal fat mass (10) and the 4:3 diet (4 days feeding:3 days fasting) reduces gonadal and inguinal WAT mass in male mice fed a low-fat or high-fat (HFD) chow, only reducing adipocyte size in HFD-fed mice (43). These different outcomes are likely to reflect the more rigorous nature of ADF and the 4:3 diet and the consequent caloric restriction. It is intriguing that this increase in inguinal and marrow adiposity is accompanied by a marked elevation in circulating FFAs, especially in males, an observation that would normally be associated with increased lipolysis and reduced adipocyte size.

### Broader lipid storage

Given this evidence of elevated lipid availability and rearranged storage in WAT, we explored whether disrupted lipid handling was more widespread. This proved to be the case. The 5:2 diet not only elevated interscapular BAT mass, but the accompanying histological and biochemical evidence of enhanced lipid storage (especially of saturated fatty acids) suggests that this may result from suppressed thermogenesis. This is supported by our quantification of *Ucp1* mRNA, the classic marker for thermogenesis in BAT, with expression of *Ucp1* mRNA in 5:2-fed females being only 77% of that in AL-fed females after only 3 weeks on the diet. Again, this contrasts with previous reports that IF-induces thermogenesis (12, 40), with the 2:1 and 4:3 diets and ADF elevating *Ucp1* expression and reducing lipid storage in BAT while elevating the process of beiging in WAT (44). However, it should be noted that these more severe forms of IF only exerted these effects in mice fed an HFD (12, 40) but were unable to do so in more obesogenic environments (45). Thus, since elevating thermogenesis in BAT dramatically reduces circulating lipids (46), the reduction in lipid utilization we report in BAT will be likely to contribute to the elevation in circulating FFAs, thereby increasing availability for storage in other sites, raising the prospect of wider ectopic lipid accumulation.

Our data indicate that while liver weight was not significantly affected, the 5:2 diet elicited sex-independent and sex-specific changes in the hepatic lipid profile that, when combined, only resulted in a minimal increase in overall lipid storage. Previous studies have reported that male mice receiving HFD in an ADF pattern showed reduced hepatic steatosis (47, 48), while the 2:1 diet was unable to prevent hepatic steatosis in the highly obesogenic environment of HFD-fed leptin-deficient *ob/ob* mice (45). In addition, it has been reported that prolonged 5:2 intervention improved liver lipid parameters in participants with non-alcoholic fatty liver disease (49), without changing LDL or HDL cholesterol (50).

### Evidence of endocrine regulation

The impact of the 5:2 diet on marrow adiposity provides important mechanistic information. Marrow adipocytes are unique, expressing both GHSR (25) and the ghrelin activating enzyme ghrelin O-acyl transferase (51), rendering them responsive to the direct adipogenic influence of both acylated (34) and unacylated ghrelin (51). In addition, they are highly sensitive to the lipolytic and anti-adipogenic action of GH (52), the inhibitory influence of both leptin (53) and the thyroid hormones (54) on differentiation, and the complex, senescent influence of glucocorticoids (55, 56).

The role of ghrelin in mediating the impact of the 5:2 diet is confirmed by our observation that the elevation in marrow adiposity in males is abolished in adolescent GHSR-null mice and reversed in adult GHSR-null mice. However, while the reduction in marrow adiposity induced by the 5:2 diet in females is also abolished in the absence of GHSR, the fact that these responses to the 5:2 diet are opposite in males and females suggests the contribution of an additional mechanism. It is remarkable that the sex-specific impact of the 5:2 diet on marrow adiposity accurately reflects circulating corticosterone levels, suggesting a contribution of the powerful adipogenic action of the adrenal glucocorticoids (57). It is important to note that the measured corticosterone values in both AL-fed and 5:2-fed mice (70-110 ng/mL) fall within the normal circadian range for mice (50-160 ng/mL) and were an order of magnitude lower than that seen following restraint stress (800-1200 ng/mL) (58). In a broader physiological context, these changes in marrow adiposity, especially in males, could have negative consequences for osteoblast activity and bone health and this requires further investigation.

GH is profoundly lipolytic, and our demonstration of accelerated skeletal growth and elevated plasma FFA concentration, especially in males, is consistent with activation of the GH-IGF-1 axis. Normally, this would be expected to result in a reduction in adipocyte size and WAT mass. However, inguinal and marrow adipocytes represent the two most sensitive depots in mice to the direct actions of ghrelin (25, 34, 51), lipid stored in marrow adipocytes being the last reservoir to be utilized during periods of fasting (59). Thus, while the 5:2 diet may enhance GH-induced lipolysis, the bi-weekly surges in ghrelin secretion induced by the two 24 h fast periods appear to defend stored lipid (24) in these ghrelin-sensitive locations.

In addition, while we have not explored the impact of deleting GHSR on the 5:2 diet-induced increase in BAT mass, it is clear from the literature that ablating GHSR, especially in BAT itself (60) augments thermogenic capacity and Ucp1 expression in aging mice (61, 62). Thus, it appears that the majority of the growth and metabolic consequences of the 5:2 diet are likely to be mediated by the twice weekly surges in ghrelin secretion and the consequent activation of GHSR. Interestingly, while we report some subtle changes in the hepatic lipid profile, in the absence of GHSR expression in liver (32), overall lipid storage is not increased.

The most prominent functional exception to this ghrelin-dependency is the weight loss impact of the 5:2 diet in adult mice, which is maintained in GHSR-null males. This corroborates a previous report that the metabolic benefit of caloric restriction in older male mice occurs independently of ghrelin (63). These findings may reflect an age-dependent decline in ghrelin production (64) and sensitivity, middle aged (7-month old) rats showing reduced weight-gain responses to ghrelin treatment (65). This decline is also reflected in lower circulating ghrelin levels in older adult humans (66).

### What might this mean for humans?

Given the numerous reports of the benefits of the 5:2 diet as a weight loss strategy in humans (5, 7–9) our mouse data are rather surprising. The difference could reflect the fact that the majority of these human studies have been performed in the context of overweight and obese participants, whereas our study was conducted in non-obese mice. However, since ADF has also been reported to induce metabolic benefits in healthy non-obese individuals (67), the reason for this difference may lie elsewhere. While the higher metabolic demands of thermogenesis in mice are an obvious species difference that may prevent similar changes in BAT mass being observed in humans, some consideration needs to be given to the precise details of the 5:2 strategies employed. In contrast to our mouse study in which food intake was completely removed during the fast days and AL feeding was permitted on re-feeding days, many human studies permit some consumption on fast days (68) and limit intake on refeeding days (69). That said, while rebound over-consumption on AL non-fast days has been reported in human volunteers, this does not appear to result in elevated overall caloric intake in humans (70), or in mice in the current study.

## Conclusion

While the weight loss benefits of the 5:2 diet in humans are associated with more favorable ratings than caloric restriction (71), our studies in mice add another note of caution to that made in a recent metanalysis of human studies, which concluded that intermittent fasting results in little to no difference in weight loss (72). In addition to previous reports that the 5:2 diet and ADF fail to induce hippocampal neurogenesis or improve spatial memory in mice (15, 73), our current study indicates that it may only be effective as a weight loss strategy in males. In this context, activation of the GH-IGF-1 axis promotes skeletal growth and elevates circulating FFAs, but lipolysis is countered by the bi-weekly surges in ghrelin, that defend stored lipid in ghrelin-sensitive inguinal and marrow WAT (24, 25, 34), ultimately leading to fat redistribution. Indeed, the elevation in lipid storage in the context of weight loss implies a decline in lean mass, a phenomenon reported to occur in humans when the 5:2 diet is combined with sprint interval training (74). Thus, it appears that the 5:2 diet has broader endocrine and metabolic consequences in males that may not be desirable in a healthy weight-loss strategy, while, in non-obese female mice, at least, these negative outcomes occur in the absence of the desired weight loss.

## Data Availability

The raw data supporting the conclusions of this article will be made available by the authors, without undue reservation.

## Footnotes

1
https://imagej.nih.gov/ij/download.html
